# The Differential Profile of Social Anxiety Disorder (SAD) and Avoidant Personality Disorder (APD) on the Basis of Criterion B of the DSM-5-AMPD in a College Sample

**DOI:** 10.21315/mjms2019.26.5.7

**Published:** 2019-11-04

**Authors:** Azad Hemmati, Sahar Rezaei Mirghaed, Fateh Rahmani, Saeid Komasi

**Affiliations:** 1 Department of Psychology, University of Kurdistan, Sanandaj, Iran; 2 Neurosciences Research Center, Research Institute for Health Development, Kurdistan University of Medical Sciences, Sanandaj, Iran

**Keywords:** Alternative Model for Personality Disorders, avoidant personality disorder, maladaptive traits, psychopathology, social anxiety

## Abstract

**Background:**

The present study was conducted to determine the differential profile of social anxiety disorder (SAD) and avoidant personality disorder (APD) based on dimensional diagnosis in criterion B of the DSM-5 Alternative Model for Personality Disorders (DSM-5-AMPD) in a college sample.

**Methods:**

Samples of this cross-sectional study included 320 (23.08 ± 2.66 years; 57% female) college students in western Iran during February 2015 to December 2017. Liebowitz-social anxiety scale, PID-5, SCID-II, SCID-II-SQ and diagnostic interview for SAD were the tools. The data were analysed using Pearson correlation and multiple linear regression analysis.

**Results:**

Forty-three and 38 participants met criteria for SAD alone and APD, respectively. Five main domains of PID-5 could explain 29% and 54% of the variance of SAD and APD, respectively. Facets of negative affect, detachment, antagonism, disinhibition, and psychoticism could explain 25% versus 43%, 26% versus 54%, 7% versus 27%, 21% versus 41%, 13% versus 30% of the variance of SAD and APD, respectively.

**Conclusion:**

SAD and APD probably refer to two distinct mental states having prominent anxiety, emotional instability, and interpersonal pattern of avoidance and detachment of challenge. SAD is a simple form of mental disturbances with anxiety in its core features; although, APD is possibly referring to more complicated psychopathology.

## Introduction

Social anxiety disorder (SAD) with an early onset, a chronic and severe inter/intrapersonal impairments, and high costs in several different aspects (1) and with second rate prevalence among anxiety disorders in adults and its 13% prevalence in community sample, located in third order place of psychiatric disorders (2), that is positively correlated with shyness (3). Kendler and Prescott (4) in their longitudinal etiological twin study referred it to genetic (17%), shared environmental (15%) and individual-specific environmental (68%). It is almost doubtless that personality is closely linked in most of the psychopathologic factors (5). As Bruch and Cheek (6) had proposed a personality based model for the explanation of social phobia, the other research literatures also consist many personality components and factors that are connected to SAD, such as hostility and aggressiveness (7), big five personality traits (8), emotion regulation strategies (9), interpersonal relationships (10), self-criticism (11) attractiveness or desirability (12).

Hofmann et al. (13), in their review study of 25 years of research on SAD, has shown that diagnostic criteria have been tremendously depending on number and types of feared social situations. Watson (14) has mentioned two key taxonomic problems for the current nosology of anxiety disorders: comorbidity and heterogeneity, and reflectively proposed a quantitative hierarchical distress and fear disorder model for the reconceptualisation of them. In results social phobia placed as one subset of fear disorders category. Sellbom et al. (15) have furthered the Watson and Clark model and supplemented the temperament markers (demoralisation, dysfunctional negative emotions, and low positive emotions) to it. In the same way, some other researches have considered the emotion regulation of anxiety disorders (16, 17). However, despite these valuable and constructive efforts, there is potential heterogeneity in the exhibition of SAD symptoms for different cases (18), as it was before.

In former studies (19, 20), the most salient co-occurrence, that has seen, was between avoidant personality disorder (APD) and SAD, for instance Lampe and Sunderland (21), in their re-analysing of a large epidemiologic study, have noticed that there is no enough evidence about clarified difference between SAD and APD, except that dysfunctions in APD is more than SAD. Also, some socially anxious individuals have exhibited characteristics quite different than the prototypical person with SAD (22). These lacks of major discriminants between them convey that they are the same, but APD has somewhat more severe symptoms (23, 24). Cox et al. (19) have reported a considerable amount of co-occurrence between generalised SAD and APD and mentioned that the probability of locating in diagnosis category of APD increased with generalised SAD symptom severity. Their national epidemiologic study has illustrated that generalised SAD and APD are basically along a continuum (19). But, determining the severity of symptoms, frequently has neglected (25). Furthermore, results have shown SAD cases have had some dysfunctions in their personality traits and much more comorbidity with personality disorders (PDs), in particular with an APD (26). According to Reich, personality traits should not be ignored in the formulation of social anxiety assessment, since SAD cases probably have personality pathology that exhibits in anxiety (27). Concentrate on personality traits for attaining to an extensive understanding of likely reasons of the co-occurrence between PDs (such as APD) and ADs (such as SAD) may inform the classification activities and may also contribute to the conclusion of etiological mechanisms (28).

Several studies have provided evidence of the association between anxiety disorders and personality traits (29). This association has reported in particular with SAD, for instance in nonclinical samples, it was significantly related to low neuroticism and high extraversion (8, 30). Growing evidence involve an unusual group of SAD subjects which they manifested the impulsive traits, such as disinhibition, risk-seeking, conflicts, disruptive and exploratory behaviors (18, 31). For this reason, some researchers such as Krueger proposed a unified model of personality, personality disorders, and clinical disorders (32).

Various studies showed personality traits accompanying various mental disorders, through which distinguishable patterns of covariance are seen across psychopathologies (33). Personality traits may change processes and outcomes of treatment of mental disorders (34), for instance; comorbidity of PDs and ADs has followed with negative sequels in treatment outcomes (35). The categorical approach of DSM to SAD and APD continually has overlooked some diagnostic components which have been linked to a common externalising continuum associated with the pathologic trait domains. While DSM-5 section III alternative model for personality disorders (AMPD), instead, has considered some specific maladaptive traits (as criterion B). These traits are anxiousness is an aspect of the negative affectivity domain and withdrawal, anhedonia and intimacy avoidance are aspects of the detachment domain (36).

Hopwood et al. (37) reported significant positive correlations between APD and all of the trait domains of the AMPD of DSM-5. According to Welander-Vatn et al. (38), APD has a positive association with neuroticism, and negative correlations with extraversion, openness to experience, agreeableness and conscientiousness. Although, SAD has a positive association with neuroticism, and it is negatively associated with extraversion, agreeableness, and conscientiousness; and no statistically significant correlation with openness to experience. Considering the traits separately, they found that APD and SAD were differentially related to extraversion, openness to experience, and agreeableness. In sum, all the negative correlations were stronger with APD. It seems that APD and SAD are different manifestations of one disorder, then can we ask ourselves critically, whether any of these disorders belong in the anxiety disorders or personality disorders, singularly. Therefore, a dimensional assessment of personality traits is valuable, whether a SAD patient has an APD or not. In one hand, traits variances can explain differences between SAD subgroups, (39) in the other hand, assessment of comorbid pathologic traits would be helpful if therapists focus on specific maladaptive traits through the treatment process.

The aim of this study is the response to a basic question, whether would be more specifying for any of APD and SAD if categorical diagnosis replaces with severity determining as a dimensional diagnosis? In other words, it would be advantageous for differential diagnosis of them, if the pathologic traits are considered in the diagnosis of APD. Because it can be predicted that APD cases would have more severe pathological traits than the SAD ones.

## Materials and Methods

### Participants

The sample was 385 students (216 female) of the clients’ list (1,603 people) of psychological services clinic at University of Kurdistan who complained about anxiety and have had referred already (from February 2015 to December 2017). They voluntarily participate in this project, in echoing to researchers’ message and electronic recall. After the description of the study, they accepted to participate and wrote informed consent and responded to all measures. Finally, 320 cases’ protocol (181 female ~57%), was diagnosed valid for data analysis. The range of age for the final sample was 18 to 34 years (mean = 23.08; SD = 2.66). One hundred and sixty individuals, who had shown anxiety in social situations on basis of the measures results, participated in two interviews in addition to questionnaires that were utilised for diagnosing of APD and SAD. Forty three participants met criteria for SAD alone (7 male, 36 female); and 38 participants met criteria for APD (23 male, 15 female).

### Research Measures

#### Persian version of personality inventory for DSM-5 (PID-5)

A 220-item self-report inventory that developed to assess the pathological traits (in 5 domains and 25 facets) for criterion B of the AMPD in DSM-5-Section-III (40). A complete list of facets for each domain can be found in Table 1. Item responses are based on a Likert scale ranging from 0 to 3. In the present study, the Krueger and colleagues’ (40) algorithm to compute the score of five domains was used. According to them, computing of five domains is based on the average of the three primary facets of any domain which are mentioned as note (c) of Table 1. The translation process of PID-5 was on the basis the translation/back-translation procedure. First, the PID-5 was independently translated into Persian that this process is done by a four-member team that included two English language specialists, a psychologist who was fluent in English and a psychometrics specialist (the first authors). Then, the final Persian version was given to a professional translator for back-translation to English without any information about the original version. The developed English back-translation was sent to developers of the PID-5 for reviewing. Finally, ten items (i.e. 2, 19, 25, 51, 73, 86, 129, 152, 165, 200) of the latest version were different that translators modified them under the supervision of the first author of the English original PID-5. Cronbach’s alphas of Persian version in the 320 samples of current study for the PID-5 domains were 0.89 (disinhibition), 0.93 (detachment and negative affectivity) and 0.94 (antagonism and psychoticism). Also, Cronbach’s alphas for the 25 trait facets were acceptable, ranging from 0.70 to 0.94 that reported in Table 1.

#### Persian version of the Liebowitz social anxiety scale-self-report (LSAS-SR)

A self-report that includes 24 items to assess fear and avoidance in a range of social and performance situations (41). The LSAS-SR has shown high internal consistency (Cronbach’s alpha = 0.95) and strong convergent and discriminant validity among SAD individuals (42). The Persian version of LSAS-SR has been prepared by employing a robust empirical translation/back-translation procedure (43). In the study of Atrifard et al. (43), Persian LSAS-SR has shown acceptable test-retest reliability (0.76 to 0.84), also has had an adequate Cronbach’s alpha (0.73 to 0.93). In addition, its total score has shown a satisfactory convergence (*r* = 0.69) with a total score of Connor’s social phobia inventory (44). Cronbach’s alpha in the current sample was 0.86.

#### The structured clinical interview for DSM-Axis-II

A semi structured interview that assesses the 10 DSM–IV/DSM-5 PDs. Each PD criterion is scored using a 0 (absent), 1 (subclinical) or 2 (present) rating (45). In the current study, we used DSM-5 Section II APD dimensional scores only (DSM-IV-TR) (46); translated to Persian and adapted by Mohammadkhani et al. (47) and DSM-5; translated by Rezai et al. (48).

#### Persian version of structured clinical interview for DSM-Axis II-screening questionnaire (SCID-II-SQ)

A questionnaire with 119 closed questions that match the main questions in the SCID-II interview (49). All items assessing the presence (by yes) or absence (by no) of specific symptoms across the spectrum of PDs. Both the SCID-II and SCID-II-PQ have been translated to Persian and adapted for Iranian population (47, 50). Only the first seven items (item 1 to 7) which assess the APD, were administrated in this study. Cronbach’s alpha of this scale was 0.60.

#### Persian version of Diagnostic Interview based on the DSM-5 Criteria for SAD

An interview that includes all the ten items of A to J diagnostic criteria for SAD (DSM-5; translated by Rezai et al. (48) that were utilised for diagnosing of SAD, in this study.

### Procedures

All participants were administered the Persian (Farsi) translation of three measures: PID-5 (40); LSAS-SR (41)); and SCID-II-SQ (49); 160 sample of them who complained about anxiety, also participated in the Persian version of structured clinical interview for DSM-Axis-II (45); and the Persian version of diagnostic interview based on the DSM-5 criteria for SAD (48), that were utilised diagnosing of APD and SAD, respectively. Three interviewers were M.A. students who had been trained during a six-months course by the third author and rated by the first author.

### Data Analysis

At first, the preliminary analyses (such as eliminating the invalid protocols, data screening in considering of missing (for the case with less than 10% missing values, utilised the mean series method) and outlier values, as well as assumptions exploring) was done. Afterward, two separate multiple regression analysis (ENTER method) utilised to study of the linear regression model of pathological traits (domains and facets) as predictors and SAD and APD as criterion variables. Also, zero-order bivariate and semi-partial correlations were calculated as preliminary analyses for the regression analysis. In addition, these results will help to make clear the details for comparison of two groups.

Furthermore, the general linear model repeated measures (GLMRM) was used for differential profile analysis (51) between SAD and APD groups, on basis of T scores (mean = 50, SD = 10) in the 5 domains and 25 facets. In this study, profile analysis was applied as a special application of multivariate approach to repeated measures to a situation where there are several dependent variables all measured on the same scale at one time. All statistical analyses were performed using the IBM-SPSS software-version 24.

## Results

### Pathological Traits (Domains/Facets) as Predictor of SAD

Both zero-order bivariate and semipartial correlations between the SAD and the DSM-5 pathological traits regarding facets and domains are reported (Table 1). The SAD had significant positive zero-order correlations with the domains, except for antagonism. While semipartial correlation was significant between the SAD with negative affect, detachment and antagonism. Moreover, the zero-order correlation between SAD with the 23 trait facets was positively significant, with the risk-taking trait was negative significant, and with the manipulativeness and the grandiosity ones was not significant. Whilst the SAD showed significant positive semi-partial correlation, only with distractibility, perceptual dysregulation, anxiousness, withdrawal, depressivity, callousness and deceitfulness, respectively; while showed significant negative semi-partial correlation with the risk-taking, and manipulativeness. Furthermore, results of multiple regression analysis (through ENTER method), showed the model which containing the seven traits of negative affect domain has explained 25% variance in the SAD (*R*^2^ = 0.25, *F* = 15.17, *P* < 0.001); the model which containing the five traits of detachment domain has explained 26% variance in the SAD (*R*^2^ = 0.26, *F* = 21.89, *P* < 0.001); the model which containing the five traits of antagonism domain has explained 7% variance in the SAD (*R*^2^ = 0.07, *F* = 4.49, *P* < 0.01); the model which containing the five traits of disinhibition domain explained 21% variance in the SAD (*R*^2^ = 0.21, *F* = 16.98, *P* < 0.001); the model which containing the three traits of psychoticism domain explained 13% variance in the SAD (*R*^2^ = 0.13, *F* = 15.11, *p* < 0.001); and the model which contained the all of five domains has explained 29% variance in the SAD (*R*^2^ = 0.29, *F* = 25.95, *P* < 0.001). Altogether, the rank of models on basis of the percent of variance explanation was five domains model (29%), detachment traits model (26%), negative affectivity traits model (25%), disinhibition Traits model (21%), psychoticism traits model (13%), and antagonism traits model (7%). As can be seen, a considerable point is that the traits of antagonism domain had the negligible amount of variance explanted for SAD.

### Pathological Traits (Domains/Facets) as Predictor of APD

Both zero-order bivariate and semi-partial correlations between the APD and the DSM-5 pathological traits regarding facets and domains are reported (Table 1). The APD had significant positive zero-order correlations with all the domains. While semi-partial correlation of APD was significant only with the negative affect, detachment and disinhibition. Furthermore, the zero-order correlation between APD with the 23 traits was positively significant, but with the risk-taking trait was negative significant and with the grandiosity was not significant. The APD showed significant positive semi-partial correlation, with the perceptual dysregulation, withdrawal, distractibility, deceitfulness, callousness, irresponsibility, anxiousness, depressivity, restricted affectivity, grandiosity, separation insecurity, and rigid perfectionism, respectively; while showed significant negative semipartial correlation with the manipulativeness, risk-taking, as well as unusual beliefs and experiences, respectively. Plus, results of multiple regression analysis (ENTER method), presented that the model which containing the seven traits of negative affectivity domain has explained 43% variance in the APD (*R*^2^ = 0.43, *F* = 33.39, *P* < 0.001); the model which contains the five traits of detachment domain has explained 54% variance in the APD (*R*^2^ = 0.54, *F* = 74.51, *P* < 0.001); the model with five traits of antagonism domain as predictors, has explained 27% variance in the APD (*R*^2^ = 0.27, *F* = 23.55, *P* < 0.001); the model including the five traits of disinhibition domain explained 41% variance in the APD (*R*^2^ = 0.41, *F* = 43.84, *P* < 0.001); the model which containing the three traits of psychoticism domain explained 30% variance in the APD (*R*^2^ = 0.30, *F* = 45.18, *P* < 0.001); and the model which contains the all of five domains has explained 54% variance in the APD (*R*^2^ = 0.54, *F* = 72.18, *P* < 0.001). In total, the sort of models in considering the percent of variance explanation was detachment traits model (54%), five domains model (54%), negative affectivity traits model (43%), disinhibition traits model (41%), psychoticism traits model (30%), and antagonism traits model (27%). All told, the noteworthy result is that the amounts of APD variance explanation by all of these models are more than SAD ones.

### Pathological Traits (Domains/Facets) as a Discriminator of SAD and APD

The findings can be considered from two levels. The first is the pathological trait domains. According to the results both SAD and APD are very much similar in negative affect and detachment domains; the negative affectivity domain show a significant semi-partial correlation with SAD (0.24; *P* < 0.001) and APD (0.21; *P* < 0.001) similarly, whereas, detachment domain has a more semi-partial correlation with APD (0.32; *P* < 0.001) than SAD (0.24; *P* <0.001). However, antagonism and disinhibition domains have a complicated presence in both these disorders. The antagonism has a negative semi-partial correlation with SAD (−0.12; *P* <0.05). However, it has no significant relationship with APD. In contrast, disinhibition domain has a mild significant relationship with APD (0.13; *P* < 0.05) and no relationship with SAD. Finally, none of them have a significant relationship with psychoticism.

The results of multiple regression analysis (ENTER method), indicated that the model which contains the five domains has explained 29% variance in the SAD (*R*^2^ = 0.29, *F* = 25.96, *P* < 0.001); results of multiple regression analysis, presented that the model which containing the five domains has explained 54% variance in the APD (*R*^2^ = 0.54, *F* = 72.19, *P* <0.001). In other words, it can be concluded that the pathological trait domains, have a much stronger prediction power for APD than for SAD.

The second is pathological trait facets level. First of all, there are a parallel presence of six trait facets for both of SAD and APD; anxiousness (0.26 versus 0.24; *P* < 0.001), withdrawal (0.26 versus 0.29; *P* < 0.001), distractibility (0.28 versus 0.27; *P* < 0.001), perceptual dysregulation (0.28 versus 0.39; *P* < 0.001), manipulativeness (−0.15 versus −0.17; *P* < 0.01) and risk-taking (−0.19 versus −0.15; *P* < 0.01). Furthermore, there are four trait facets more significant for APD than for SAD; depressivity (0.23; *P* < 0.001 versus 0.15; *P* < 0.01), callousness (0.26; *P* < 0.001 versus 0.14; *P* < 0.05), deceitfulness (0.26; *P* < 0.001 versus 0.11; *P* < 0.05), and irresponsibility (0.25; *P* < 0.001 versus 0.02; *P* > 0.05). There is no trait facet with a significant relationship with SAD without a significant relationship with APD. Conversely, there are five facets with a significant relationship with APD without a significant relationship with SAD; separation insecurity (0.12; *P* < 0.05), restricted affectivity (0.16; *P* < 0.01), grandiosity (−0.14; *P* < 0.05), rigid perfectionism (0.12; *P* < 0.05), and unusual beliefs and experiences (0.16; *P* < 0.01).

There also are some important conclusions according to semi-partial correlation results. Some of the correlations appear very high in zero-order correlation but decrease notably in semi-partial correlation. Those false correlations indicate the most powerful presence of other trait facets. So, the significant relationship of emotional lability, hostility, perseveration and submissiveness with SAD and APD were because of anxiousness; the significant relationship of anhedonia, intimacy avoidance and suspiciousness with SAD and APD were because of withdrawal and depressivity; the significant relationship of attention seeking, with SAD and APD was because of callousness and deceitfulness; the significant relationship of impulsivity with SAD, was because of distractibility, and with APD was because of irresponsibility and distractibility; the significant relationship of eccentricity with SAD and APD was because of perceptual dysregulation.

### Profile Analysis

The first aim of this analysis was to figure out whether the two groups (43 SAD and 38 APD participants) have parallel profiles in any of the 5 domains and 25 facets. The test of parallelism when using the profile approach to univariate repeated- measures ANOVA, is the test of interaction (51). Thus, both of the parallelism of profile in 5 domains and 25 facets of SAD and APD groups were tested by investigating of the group*domains and group*facets interaction to find out that two groups have the same pattern of highs and lows on the 5 various domains and 25 facets measured by the PID-5. The second aim of this section of analysis was to examine what group (SAD or APD), on average, score higher on 5 domains and 25 facets than another, whether or not groups produce parallel profiles. For this purpose, the overall difference among 5 domains and 25 facets (see section 3.3.4) of SAD and APD profiles were explored by analysing the between-subjects’ main effects in repeated-measures ANOVA. However, profile analyses require all measures with the same scaling of scores (50). For this reason, the standardised values of all 25 facets and 5 domains were transformed to *T* scale (*M* = 50, SD = 10).

### Parallelism in 5 Pathological Trait Domains Profiles of SAD versus APD Group

The results of univariate repeated measures (ANOVA) showed that group*domains interaction in within-subjects effects analysis, is not significant (Huynh-Feldt F _(3.63, 287.09)_ = 2.42, *P* = 0.055, Partial *η*^2^ = 0.03). This result shows that SAD and APD groups have produced parallel profiles in 5 pathological trait domains (see the results belong to profile 1 in Table 2).

### Overall Difference among 5 Pathological Trait Domains Profiles of SAD versus APD Group

**Figure 1:**
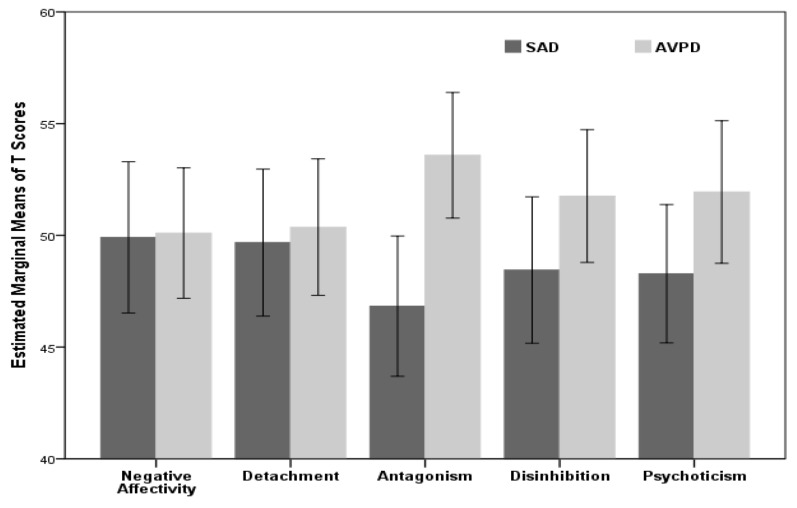
The 5 domains’ estimated marginal means of *T* scores for SAD versus APD group

The results of univariate repeated measures (ANOVA) showed that main effect (groups) in between-subjects analysis, is not significant (*F*_(1, 79)_ = 3.36, *P* = 0.071, Partial *η*^2^ = 0.04). In other words, in addition to that two groups didn’t show parallel profiles, they have not a significant overall difference among 5 domains of pathological traits, on average (see the results of profile 1 in Table 2 and Figure 1).

### Parallelism in 25 Pathological Trait Facets Profiles of SAD versus APD Group

The results of univariate repeated measures (ANOVA) showed that group*facets interaction in within-subjects effects analysis, is significant (Huynh-Feldt *F*_(16.18, 1277.96)_ = 1.89, *P* = 0.017, Partial *η*^2^ = 0.023). This means that SAD and APD groups have produced non-parallel profiles in 25 pathological trait facets (see the results of profile 2 in Table 2).

### Overall Difference among 25 Pathological Trait Facets Profiles of SAD vs. APD Group

**Figure 2:**
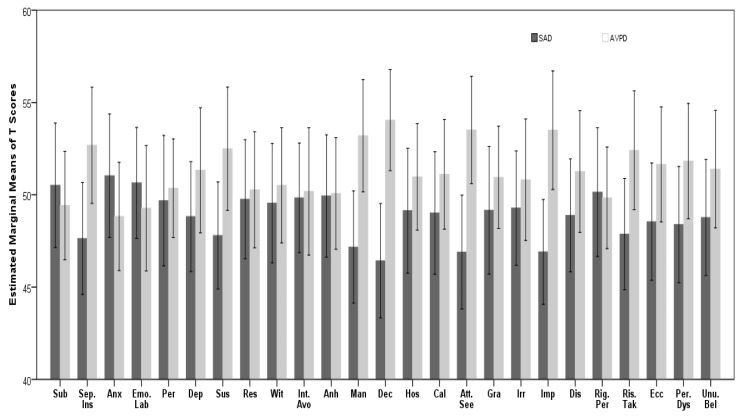
The 25 pathological traits’ estimated marginal of *T* scores for SAD versus APD group

The results of univariate repeated measures (ANOVA) showed that main effect (groups) in between-subjects analysis, is significant (*F*_(1, 79)_ =4.49, *P* = 0.037, Partial *η*^2^ = 0.054). In other words, in addition to that two groups showed parallel profiles, they have a significant overall difference among 25 facets of pathological traits, on average (see the results belong to profile 2 in Table 2 and Figure 2).

## Discussion

Generally, it is can be concluded that the diagnostic and prediction of the value of these traits are much more for APD than for SAD. The SAD group may have any personality profile; however, APD clinical profile regarding trait facets is more specific and coherent. There are some interpretations from the results that must be considered about the subjects. Intimacy avoidance is not a real trait in SAD or APD, while, their real traits are depressivity and withdrawal. Also, irresponsibility and distractibility lead to a person’s inner experience to be impulsivity; what s/he may experience and the others do not realise it. Possible hostility of the subjects, their emotional lability and submissiveness are the results of their intense anxiety in fact.

Historically, the possible association of APD and SAD was challenging. There are studies that presented similar personality profile for both disorders (52, 53). But many studies support the assumption of severity continuum, through which APD is the intense form of SAD (20, 54). The results are also congruent with Lampe (54); the more problematic clinical features of APD contrasting SAD. Even though it is contradicted to the fact that all APD cases have not the diagnostic criteria of SAD, the results of the current study are congruent with such a point of view. Some other findings of APD are also approved by the current study; including the high risk for depression (20) and disturbed interpersonal functioning (20, 23). The results are agreed with the lower rate of conscientiousness in APD group according to NEO-PI-R, contrasting SAD, and a similar level of introversion (20). It is evident from the results that personality functioning is more impaired in APD group. There are many other studies uncovered such dysfunction (24).

The results of the current research are highly congruent with Hopwood et al. (37) in term of significant positive correlations between APD and all the trait domains of the AMPD of DSM-5. It also has a considerable congruence with Welander-Vatn et al. (38), which have shown a positive association between APD and neuroticism (comparable to negative affectivity), and negative associations with extraversion (comparable to detachment), openness to experience (comparable to psychoticism), agreeableness (comparable to antagonism), and conscientiousness (comparable to disinhibition). They also found SAD to be positively associated with neuroticism, negatively correlated with extraversion, agreeableness, and conscientiousness, and having no significant association with openness to experience. The most important similarity is that there were stronger negative correlations with APD for all the trait facets and domains.

## Conclusion

SAD and APD probably refer to two distinct mental states having prominent anxiety, emotional instability and interpersonal pattern of avoiding and detachment challenge) (54). However, people with SAD show a more evident facet of anxiety. According to the results, APD is possibly referring to more complicated psychopathology and SAD is a simple form of mental disturbances with anxiety in its core features. About APD, it can be expressed that some important theoretical explanations can be adopted according to the results. These include polarity (the simultaneous presence of opposite personality trends or processes) and different mental layers (something mental states or processes hidden, which may be felt and thought by the person, and something evident, which can be seen and inferred by both the person and the others). However, it is not possible to describe/explain APD according to these results. It needs more comparisons with other personality disorders.

## Figures and Tables

**Table 1:** Zero-order bivariate and semipartial correlations of personality traits (domains/facets) with the SAD and APD; accompany with multiple regression results (*N* = 320)

Personality Traits	Alpha	Multiple Regressions							

		SAD a				APD b			

		Zero-order *R*	Semi-partial *R*	Beta	*R*^2^	Zero-order *R*	Semi-partial *R*	Beta	*R*^2^
***Domains*** c					0.29***				0.54***
Negative affectivity	0.87	0.45***	0.24***	0.34***		0.61***	0.21***	0.30***	
Detachment	0.85	0.45***	0.24***	0.30***		0.63***	0.32***	0.40***	
Antagonism	0.84	0.05	−0.12*	−0.16**		0.29***	0.04	0.05	
Disinhibition	0.86	0.36***	0.02	0.03		0.59***	0.13*	0.21**	
Psychoticism	0.94	0.28***	0.01	0.01		0.47***	−0.05	−0.08	
Negative affect facets					0.25***				0.43***
Anxiousness	0.82	0.47***	0.26***	0.06		0.55***	0.24***	0.31***	
Emotional lability	0.69	0.33***	0.07	−0.04		0.45***	0.08	0.11	
Separation insecurity	0.71	0.26***	−0.03	0.34***		0.46***	0.12*	0.16**	
Hostility	0.81	0.27***	0.003	0.09		0.37***	−0.01	−0.02	
Perseveration	0.76	0.37***	0.06	0.08		0.52***	0.09	0.13*	
Restricted affectivity d	0.66	0.26***	0.08	0.09		0.38***	0.16**	0.18***	
Submissiveness	0.49	0.28***	0.05	0.00		0.35***	0.01	0.01	
Detachment facets					0.26***				0.54***
Anhedonia	0.46	0.38***	−0.01	−0.01		0.59***	0.02	0.04	
Intimacy avoidance	0.28	0.14*	−0.03	−0.03		0.16**	−0.09	−0.09*	
Withdrawal	0.85	0.46***	0.26***	0.35***		0.62***	0.29***	0.39***	
Suspiciousness	0.51	0.26***	0.01	0.01		0.44***	0.09	0.11*	
Depressivity	0.89	0.42***	0.15**	0.25**		0.65***	0.23***	0.38***	
Antagonism facets					0.07**				0.27***
Callousness	0.78	0.14*	0.14*	0.17*		0.39***	0.26***	0.33***	
Deceitfulness	0.73	0.13*	0.11*	0.17*		0.41***	0.26***	0.42***	
Manipulativeness	0.61	−0.01	−0.15**	−0.23***		0.19**	−0.17**	−0.27***	
Attention seeking	0.76	0.12*	0.09	0.11		0.21***	0.08	0.09	
Grandiosity	0.71	−0.05	−0.09	−0.10		−0.01	−0.14*	−0.16**	
Disinhibition facets					0.21***				0.41***
Irresponsibility	0.43	0.19**	0.02	0.02		0.49***	0.25***	0.30***	
Impulsivity	0.63	0.23***	0.02	0.03		0.42***	0.05	0.07	
Risk taking	0.42	−0.22***	−0.19**	−0.20***		−0.15**	−0.15**	−0.15**	
Rigid perfectionism^d^	0.78	0.17**	0.07	0.07		0.22***	0.12*	0.12**	
Distractibility	0.85	0.41**	0.28***	0.36***		0.55***	0.27***	0.34***	
Psychoticism facets					0.13***				0.30***
Eccentricity	0.92	0.21***	0.01	0.01		0.37***	0.07	0.09	
Perceptual dysregulation	0.85	0.35***	0.28***	0.40***		0.54***	0.39***	0.57***	
Unusual beliefs & experiences	0.79	0.17**	−0.06	−0.08		0.28***	−0.11*	−0.14*	

(a) Six separate multiple regressions (ENTER method) was utilised with domains/traits as predictors and SAD as criterion;

(b) Six separate multiple regressions (ENTER method) was utilised with domains/traits as predictors and APD as criterion;

(c) The five domains were computed on the basis of the average of the three primary facets of any domain: negative affect (anxiousness, emotional lability, separation insecurity); detachment (anhedonia, intimacy avoidance, withdrawal); antagonism (deceitfulness, grandiosity, manipulativeness); disinhibition (distractibility, impulsivity, irresponsibility); psychoticism (eccentricity, perceptual dysregulation, unusual beliefs and experiences) (see 39);

(d) lower scores (lack of) indicate higher domain scores;

* *P* < 0.05.

** *P* < 0.01.

*** *P* < 0.001

**Table 2:** Repeated measure ANOVA summary results for differential profile analysis of 5 domains (profile 1) and 25 facets (profile 2) between SAD and APD groups.

Profile	Test	*F* (DFs)	Sig.	*η*^2^
Profile 1 (5 domains)	Test of parallelism (domains*groups)	2.42 (3.63, 287.09)^a^	0.06	0.03
	Test of overall difference (groups)	3.36 (1, 79)	0.07	0.04
Profile 2 (25 facets)	Test of parallelism (facets*groups)	1.89 (16.18, 1277.96)^b^	0.02*	0.02
	Test of overall difference (groups)	4.49 (1, 79)	0.04*	0.05

(a) The alternative test was Huynh-Feldt with adjusted DF for not assumed sphericity (Mauchly’s W = 0.71; Approx. chi-square = 26.96; DF = 9; *P* < 0.01);

(b) The alternative test was Huynh-Feldt with adjusted DF for not assumed sphericity (Mauchly’s W = 0.00; Approx. chi-square= 680.67; DF = 299; *P* < 0.001);

* *P* < 0.05
